# A Case—Uncontrollable—No. 6

**Published:** 1862-11

**Authors:** 


AN UNCONTROLLABLE CASE—No. 6.

Rather a singular case came under our observation to-day. A gentleman called to have several teeth filled. After preparing a cavity, in the first superior molar, anterior proximal side, we attempted to fill it; and first, as the custom is, the fold of a napkin was placed about the tooth, to protect it from the saliva, but in a moment or two, the man made an effort as if to vomit, so that the napkin had to be removed; then a roll of bibulous paper was placed between the cheek and the tooth, and a small piece of spunk on the palatine side of the tooth, and overlapping the margin of the gum; these were retained lightly and carefully in place with the fingers, but in a few moments the same difficulty again occurred, when he remarked that the presence of these things seemed to make him sick: but they were again placed in and remained there, till that tooth was filled. Three lower molars were then to be filled, but it was found to be impossible to keep anything about them. About the first of these a roll of paper was kept a few moments; then it had to be removed, then to finish that one, the fingers only were kept about the tooth, and soon the same repugnance to the fingers was manifested, and the last three teeth, two inferior and one superior, were filled without anything being put into the mouth, except the plugging instrument, and then he had to close the mouth every moment or two to swallow.

Of course, it was to a considerable extent a submarine operation, but that was the best that could be done. What was the matter with the man? I do not know, unless it was irritation of the throat.

